# An efficient method to identify virus-specific TCRs for TCR-T cell immunotherapy against virus-associated malignancies

**DOI:** 10.1186/s12865-021-00455-3

**Published:** 2021-09-28

**Authors:** Lei Chen, Lianhua Dong, Yipeng Ma, Juntao Wang, Dongjuan Qiao, Geng Tian, Mingjun Wang

**Affiliations:** 1 Department of Research and Development, Shenzhen Institute for Innovation and Translational Medicine, Shenzhen International Biological Valley-Life Science Industrial Park, Dapeng New District, Shenzhen, China; 2 Department of Oncology, Shenzhen Second People’s Hospital, The First Affiliated Hospital of Shenzhen University, Shenzhen, 518035 China

**Keywords:** TCR cloning, Immunotherapy, Virus-specific TCRs, TCR transduced T cells

## Abstract

Adoptive transfer of T cells genetically engineered with a T cell receptor (TCR) is a promising cancer treatment modality that requires the identification of TCRs with good characteristics. Most T cell cloning methods involve a stringent singularization process, which necessitates either tedious hands-on operations or high cost. We present an efficient and nonstringent cloning approach based on existing techniques. We hypothesize that after elimination of most nonspecific T cells, a clonotype with high quality could outcompete other clonotypes and finally form a predominant population. This TCR identification method can be used to clone virus-specific TCRs efficiently from cancer patients and is easily adoptable by any laboratory.

**Supplementary Information:**

The online version contains supplementary material available at 10.1186/s12865-021-00455-3.

## Introduction

Viral infections cause approximately 10–12% of all human malignancies worldwide. To date, at least 8 viruses have been associated with human cancers, including Epstein-Barr virus (EBV), papillomavirus (HPV), Kaposi’s sarcoma-associated herpesvirus (KSHV), Merkel cell polyomavirus (MCV), hepatitis B virus (HBV), human T lymphotropic virus type-1 (HTLV-1), hepatitis C virus (HCV) and human immunodeficiency virus (HIV) [1]. In the life cycle of some viruses, especially EBV and HPV, virus-derived oncogenic proteins are always present in malignant cells, making them excellent targets for T cells. For example, the high-risk HPV-encoded oncoproteins E6 and E7 are essential for the generation and maintenance of HPV-related malignancies [2, 3]. All EBV-associated cancers express some EBV latency antigens (EBNA1, EBNA2, EBNA3-3C, EBNA-LP, LMP1 and LMP2) [4]. These intracellular or membrane proteins can be processed and presented on the host cell surface in the context of MHC molecules and recognized by T cell receptors (TCRs) as foreign antigens. Therefore, compared with traditional cancer therapies, adoptive T-cell therapies (ACTs) may have unique advantages in eradicating virus-associated malignancies [5, 6].

Over the past decades, ACT has achieved significant clinical efficacy in the treatment of many human malignancies [7–9]. It has also been demonstrated that the adoptive transfer of virus-specific cytotoxic T cells isolated and expended directly from tumor specimens (i.e., TILs) can mediate durable and even curative responses [10–12]. However, the widespread use of TILs has been limited by the challenge of consistently isolating autologous viral-specific TILs for each patient, a long timeframe to validate reactive TILs, limited in vivo proliferation potential of the final cell product, and a lack of standardized manufacturing processes [13–15]. In contrast, TCR gene-modified T cells can redirect nonspecific T cells against a predefined antigen and provide an attractive alternative to treat virus-related malignancies [16, 17].

The discovery of TCR was the key step in the development of TCR-T cell therapy. Several approaches have been explored to identify tumor-specific TCRs. The conventional approach involves T cell cloning through multiple rounds of limiting dilution to isolate reactive T cell single clones. This method is labor intensive and time consuming [18, 19]. Multimer- or activation marker-guided cell isolation facilitates the discovery of antigen-specific T cells, but it requires the frequency of the antigen-specific T cells to be above the detection limit of flow cytometry [20, 21]. In recent years, several single-cell-based approaches have been developed [22–24]. These approaches involve the distribution of a single T cell into 96-well or 384-well plates through flow cell sorting or droplet microfluidics, RT-PCR to amplify paired TCR α and β chains, and subsequent Sanger sequencing. Although these approaches are highly efficient, they are still expensive and not readily available in most labs.

Here, we present an efficient method to efficiently isolate virus-specific TCRs from virus-positive cancer patients. First, we found that many virus-positive patients have endogenous T cell responses against viral antigens, even though the immunogenicity of several viral antigens is low. Second, unlike the isolation of neoantigen-reactive TCRs, we only need one TCR with good properties against a predetermined viral epitope. We hypothesized that after elimination of most nonspecific T cells, a lead functional clone could outcompete other clones due to its strong proliferation potential. Therefore, we used a one-round nonstringent limiting dilution to isolate highly reactive polyclones and expanded these polyclones directly. Tetramer-positive cells were isolated from polyclones that could thrive and subjected to library preparation (5’ RACE technique [25]) and next-generation sequencing (NGS). In many cases, we retrieved only one dominant full-length Vα and Vβ chain that could pair successfully. By using this approach, we quickly isolated TCRs against EBV, HPV and CMV. All TCRs showed high transduction efficiency, good alpha- and beta-chain pairing, high avidity and potent in vitro function.

## Results

### An efficient method to isolate virus-specific TCR

**Fig. 1:**
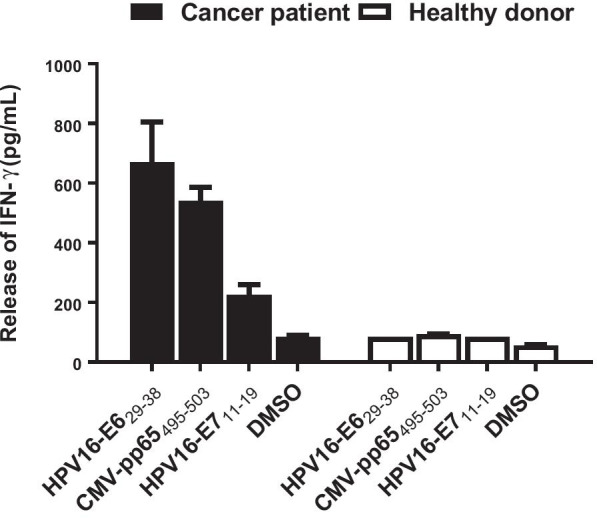
High endogenous reactivities to viral antigens of PBMCs from cancer patients. PBMCs from a healthy donor and a patient with metastatic cervical cancer were cocultured with T2 cells pulsed with indicated peptides or DMSO for 16–18 h, respectively. The release of IFN-γ in the cocultured supernatant were detected by ELISA. The IFN-γ release of PBMCs from the cancer patient were three to eightfold higher than that of PBMCs from the healthy donor. Similar results were observed in patients with metastatic melanoma and NPC

**Fig. 2:**
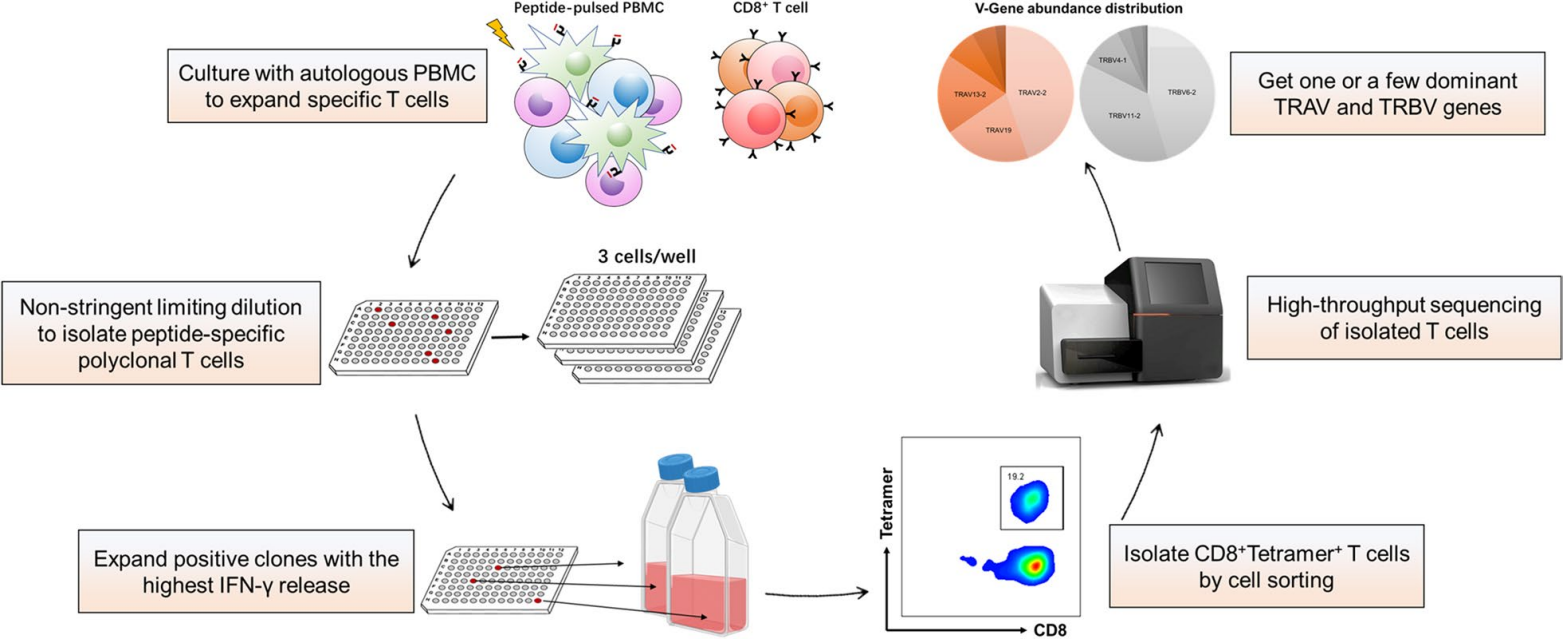
Schematic of the nonstringent TCR cloning method. CD8^+^ T cells of cancer patients were first cocultured with irradiated autologous PBMC pulsed with the target viral peptide for 7–10 days. The reactivities of T cells were tested by ELISA and T cells with high IFN-γ release were subjected to one round of nonstringent limiting dilution cloning. The second ELISA tests were performed to identify T cells that still had high IFN-γ release. The polyclonal T cells with high activities were expanded in T25 flasks. Tetramer-guided cell sorting was performed to collect 0.5–2 × 10^6^ cells for ImmunoSEQ. The TCR pair was identified by the distribution abundance of TCRα and TCRβ chains

**Fig. 3:**
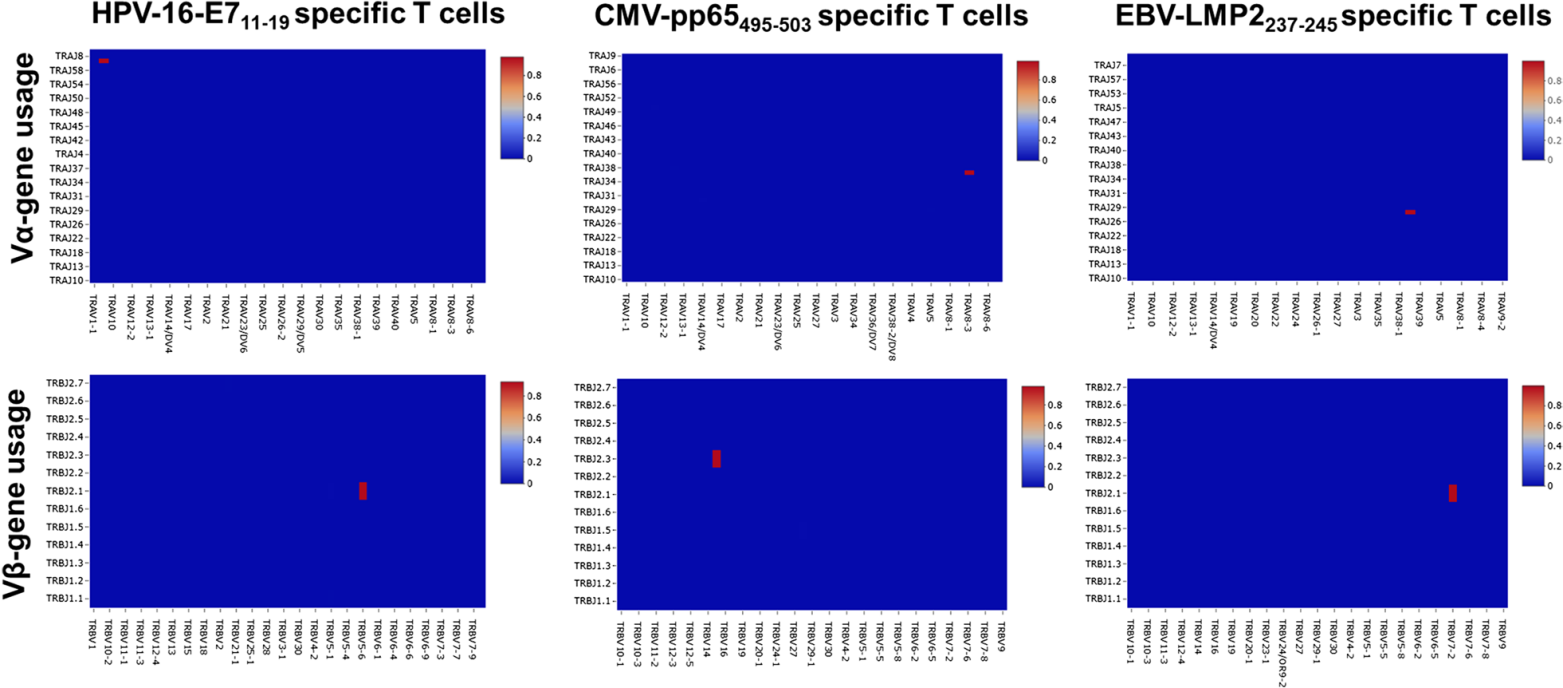
A single dominant TCRα chain and TCRβ chain were retrieved from ImmunoSEQ results. Heatmaps of TCR variable gene usage were displayed. For HPV-16-E7_11–19_ specific T cells, the top Vα accounts for 97.74% and the top Vβ accounts for 92.38%. For CMV-pp65_495–503_ specific T cells, the frequencies of the top Vα and Vβ are 91.62% and 97.15%, respectively. The frequencies of the top Vα and Vβ of EBV-LMP2_237–245_ specific T cells are 99.9% and 99.64%

Peripheral blood mononuclear cells (PBMCs) collected from HLA-A2-positive patients with different malignancies, including nasopharyngeal carcinoma (NPC), metastatic cervical cancer and melanoma were used to isolate virus-specific TCRs. Compared with PBMCs from healthy donors, PBMCs from cancer patients showed higher endogenous reactivities to distinct viral antigens (Fig. 1 and Additional file 1: Fig. S1), which indicates that (1) these epitopes are immunogenic and feasible targets for human T cells and (2) cancer patients would be a good source of virus-specific TCRs. As illustrated in Fig. 2, CD8^+^ T cells were first sensitized with irradiated, peptide-pulsed autologous PBMCs. Since the target epitopes are MHC-I restricted, we purified CD8^+^ T cells to eliminate the interference of a large number of nonspecific CD4^+^ T cells at the beginning. Then, cells from strong positive wells (release of IFN-γ, OD_450_ ≥ 1 after subtracting the control background signal) were mixed and subjected to a nonstringent limiting dilution (3 cells/well). We tested limiting dilutions with 0.3, 3 and 6 cells per well and found that when a stringent limiting dilution strategy was used (0.3 cells/well), most T cells failed to grow to a sufficient number in 2 weeks. When cloning with 3 or 6 cells/well, most cells grew enough in 2 weeks for testing reactivity. Both dilution rates gave equivalent positive wells but higher background signal when using 6 cells/well. Polyclones from strongly positive wells were selected and expanded in T25 flasks. Antigen-specific T cells were further purified through tetramer selection of polyclones that grew vigorously. Finally, a full-length library was established, and TCR chains were retrieved by NGS. Interestingly, the NGS results showed one dominant TCR (Fig. 3). For example, the sequencing results of HPV16-E7-specific T cells showed a single dominant TCR alpha chain (frequency: 97.74%) and beta chain (frequency: 92.38%), suggesting that the final T cells were monoclonal. We isolated 6 TCRs by this approach: G8 TCR targets to HPV16-E7_11–19_, A6 TCR directs to CMVpp65_495–503_, A4 TCR recognizes LMP2_356–364_, and A12, C5 and G1 recognize LMP2_426–434_. All the viral epitopes are HLA-A*02:01 restricted (Additional file 1: Fig. S2).

### TCRs generated by our approach can be successfully expressed and paired on transduced T cells

**Fig. 4:**
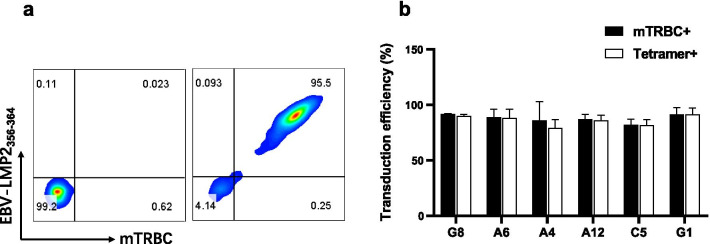
TCRs that identified by our method are successfully expressed and paired on transduced T cells. **a** A representative result of flow cytometry analysis. Plotted cells were gated on lymphocytes (SSC × FSC), live cells (7-AAD^−^), and CD3^+^ cells. The percentage of tetramer^+^ and mTRBC^+^ cells were calculated. **b** Transduction efficiency of TCRs calculated by tetramer and anti-mTRBC antibody staining. Data are representative of two independent experiments and values are expressed in mean ± SEM

To investigate whether the TCRs isolated by our approach were functional, we cloned the DNA encoding target TCRs into an MSGV1 retroviral vector. The human TCR constant regions were replaced with their mouse counterparts. One concern about the safety of TCR-transduced T cell therapy is the potential mispairing of introduced TCRs with endogenous TCRs [26, 27]. Although several protein engineering methods could be used to enhance the pairing of introduced TCRs [28], it would be best to use a TCR with inherently good pairing capacity. All TCRs isolated by using our method showed high expression and good pairing with transduced T cells, as demonstrated by the equivalent percentages of tetramer^+^ and mTRBC^+^ cell populations (Fig. 4a, b). The variable region sequences of the introduced TCRs were found to be the major determinant of the mispairing level [29]. Therefore, TCRs isolated by our method presumably have innately good characteristics.

### The selected TCRs specifically recognize viral epitopes and have high functional avidity

**Fig. 5:**
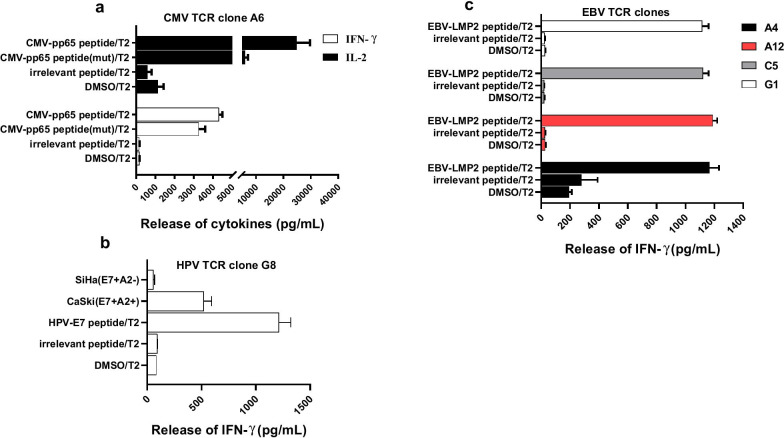
TCR-transduced T cells specifically recognized cognate peptide-loaded T2 cells. CMV-specific TCR-T cells (**a**), EBV-specific TCR-T cells (**b**) and HPV-specific TCR-T cells (**c**) were cocultured with cognate peptide, irrelevant peptide or DMSO pulsed T2 cells. The release of IFN-γ or IL-2 in the cocultured supernatant were detected by ELISA. Error bars represent the SEM for 3 technical replicates

**Fig. 6:**
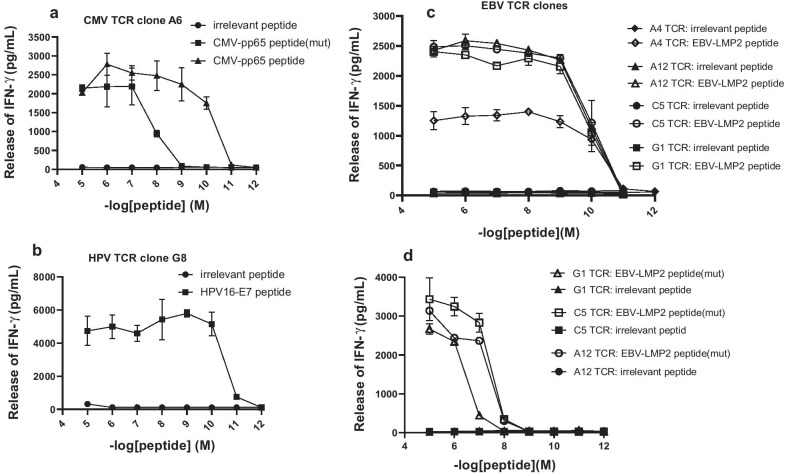
Functional avidity assay of TCR-transduced T cells. CMV-specific TCR-T cells (**a**), HPV-specific TCR-T cells (**b**) and EBV-specific TCR-T cells (**c**,** d**) were cocultured with T2 cells that pulsed with peptides at the concentration indicated on the *x-*axis. The release of IFN-γ in the cocultured supernatant were detected by ELISA. Error bars represent the SEM for 3 technical replicates

Next, we tested the functional avidity of isolated TCRs. All TCR-T cells specifically recognized cognate peptide-loaded T2 cells but not control cells (DMSO or irrelevant peptide-loaded T2 cells) (Fig. 5a–c). CMV and EBV TCR-transduced T cells (A6, A12, C5 and G1) also recognized epitopes from mutant antigens (CMVpp65_495–503_: NLVPIVATV, M499I and EBV-LMP2_356–364_: FLYALALLL, Y358C). All TCR-T cells showed a cytokine response (IFN-γ) in a dose-dependent manner and could respond to T2 cells pulsed with their cognate peptide at a concentration as low as 10 pM, indicating that all TCRs had high functional avidity (Fig. 6a–d).

### Virus-specific TCR-T cells specifically recognize and kill cancer cells expressing HLA-A2 and viral antigens

**Fig. 7:**
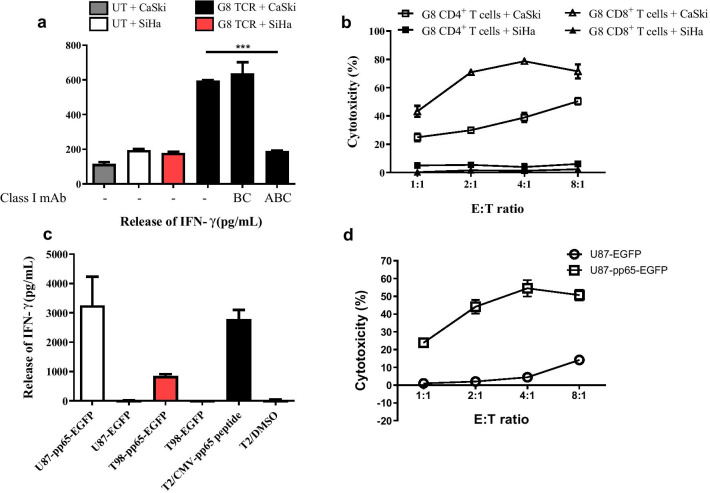
TCR-transduced T cells recognize and kill cancer cells expressing target viral antigens in vitro. **a** MHC competition assay in which the effector G8 TCR-T cells were cocultured with different target cells (CaSki: HLA-A2^+^, HPV16-E7^+^ or SiHa: HLA-A2^−^, HPV16-E7^+^) with or without blocking antibodies as indicated in *x*-axis. UT: untransduced T cells served as a negative control. **b** LDH release assay of G8 TCR transduced CD4^+^ and CD8^+^ T cells. **c** A6 TCR-T cells were cocultured with glioblastoma cell lines (U87 or T98) expressing CMV antigen or empty vector. **d** LDH release assay of A6 TCR-transduced T cells against U87 expressing CMV-pp65. The ratio of effector cells to target cells was indicated in the x-axis (E: T ratio). The percentage of cytotoxicity was calculated according to the manufacturers’ instructions

We next performed experiments to confirm that TCR-T cells specifically recognize and kill human cancer cells expressing HLA-A2 and viral antigens. G8 TCR-T cells were cocultured with the human cervical cancer cell lines CaSki (HLA-A2^+^, E7^+^) or SiHa (HLA-A2^−^, E7^+^). G8 TCR-T cells specifically reacted with CaSki but not SiHa. The recognition of CaSki by G8 TCR-T cells was significantly inhibited by antibody blockade of HLA class ABC (antibody clone W6/32) but not HLA class BC molecules (antibody clone B1.23.2), which demonstrated that target recognition was HLA-A restricted (Fig. 7a). In the lactate dehydrogenase (LDH) cytotoxicity assay, both G8 TCR-transduced CD4^+^ and CD8^+^ T cells could lyse CaSki cells at a low effector-to-target ratio (Fig. 7b), indicating that the affinity of the G8 TCR was high; thus, the recognition of pMHC was CD8^+^ independent. Although the role of CMV in glioblastoma multiforme (GBM) has been controversial, some groups have reported the presence of CMVpp65 antigen in GBM tumors as a potential target for T cell therapy [30, 31]. Therefore, we constructed two CMVpp65-expressing glioblastoma cell lines (U87-pp65-EGFP and T98-pp65-EGFP) to evaluate the in vitro function of A6 TCR-transduced T cells. A6 TCR-T cells specifically recognized CMVpp65-expressing cancer cells but not empty vector-transduced cancer cells (Fig. 7c). Similarly, A6 TCR-T cells killed U87-pp65-EGFP cells at a low effector-to-target ratio. The results showed that the selected virus-specific TCRs have potent in vitro functions (Fig. 7d).

### HPV16-E7 specific TCR-T cells suppress the progression of HPV16-E7^+^ tumor in an immunodeficient mice model

**Fig. 8:**
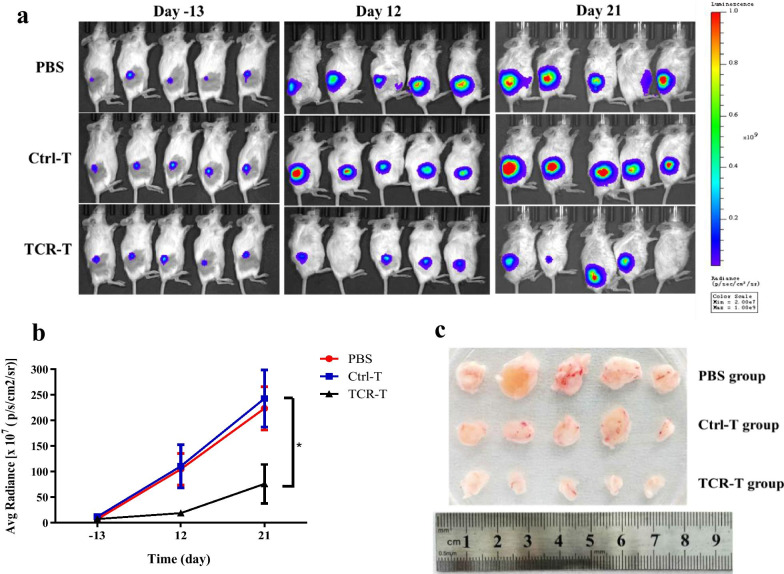
Antitumor activity of G8 TCR-T cells against cervical cancer in a mouse model. **a**–**c** NCG mice with 21-day subcutaneous CaSki-luc tumors were treated with a single intravenous injection of 4 × 10^6^ G8 TCR^+^ T cells. 50,000 IU IL-2 was given daily by intraperitoneal injection for 3 days. Ctrl-T: NCG mice were treated with untransduced T cells and IL-2. PBS: NCG mice were treated with PBS and IL-2. **a** Tumor growth was monitored by bioluminescence imaging at indicated time points. **b** Quantitative imaging data of figure A (n = 5). **c** Tumors excised from mice in each group on day 27 after TCR-T cell treatment. *p < 0.05. An unpaired two-tailed *t* test was performed

After confirming the cancer-killing ability of virus-specific TCR-T cells in vitro, we next tested the potential antitumor activity of TCR-T cells in NCG mice. Considering the availability of cancer cell lines expressing both target antigen and HLA, we chose cervical cancer as a model to evaluate the in vivo function of TCR-T cells. NCG mice implanted subcutaneously with CaSki-luc cells on day-21, were administered with phosphate-buffered saline (PBS), untransduced T cells (Ctrl-T) or G8 TCR-T cells (TCR-T) (Fig. 8a–c). A single intravenous injection of 4 × 10^6^ G8 TCR^+^ T cells was performed on day 0 with an intraperitoneal injection of IL-2 (50,000 U per day) on days 0–2. As shown in Fig. 8b, tumors continued to grow in the PBS group and Ctrl-T group, whereas the administration of G8 TCR-T cells resulted in significant suppression of the growth of CaSki-luc tumors. (*P* < 0.05). On day 27, all mice were sacrificed and the tumors were excised (Fig. 8c). Consistent with the bioluminescence imaging result, we found a marked reduction in tumor sizes of all mice in the TCR-T group compared with those in the PBS groups and the Ctrl-T group. All these results demonstrated that G8 TCR-T cells at a medium dose of 4 × 10^6^ TCR^+^ cells were able to significantly delay the progression of CaSki-luc tumors.

## Materials and methods

### Cell lines and culture media

CaSki, SiHa, U87, T98G and T2 cell lines were purchased from ATCC. Cell lines were cultured in RPMI-1640 (CaSki, SiHa and T2), or DMEM (293GP, U87 and T98G) supplemented with 10% (v/v) FBS (Lonsera), GlutaMAX (Life Technologies), 100 U/ml penicillin (Life Technologies) and 100 µg/ml streptomycin (Life Technologies). U87-pp65-EGFP, and T98-pp65-EGFP are U87 or T98-based cell lines with stable expression of pp65-GFP. These cell lines were generated by transduction of retroviral vectors encoding target proteins, and a subsequent cell sorting of GFP^+^ cells with flow cytometry. All cell lines that used in the experiments tested negative for mycoplasma. The culture medium for T cell in vitro peptide sensitization was X-VIVO™ 15 (Lonza) with 5% (v/v) human AB serum and 50 U/ml IL-2 (Peprotech). The medium used for quick expansion of T cell was X-VIVO™ 15 with 10% (v/v) FBS serum and 300 U/ml IL-2 and 50 ng/mL IL-15 (Peprotech).

### Generation of virus-specific T cell clones

PBMC were obtained by leukapheresis, prepared with density gradient centrifugation (human T lymphocyte separation medium from Dakewe Biotech), and cryopreserved. For in vitro sensitization, CD8^+^ T cells were first isolated from human PBMC using Dynabeads™ CD8 positive isolation kit (Life Technologies), and then 3 × 10^5^ CD8^+^ T cells were stimulated with irradiated, viral peptide-loaded autologous PBMC (1 × 10^6^, CD8^+^ T cell-depleted PBMC) in each well of a 24-well plate. Cultures were maintained individually for 7–10 days and replaced with fresh medium every 2 days. The reactivities of cells were tested by the IFN-γ release assay against T2 cells pulsed with cognate viral peptides or irrelevant peptides. T cell cultures with pronounced and specific recognition of the target epitope (OD_450_ ≥ 1 after subtracting control signal) were subjected to the limiting dilution cloning (3 cells/well). Specifically, T cells were stimulated with 2 × 10^6^ irradiated allogenic PBMC from three healthy donors and 30 ng/mL anti-CD3 antibody (OKT3) in the quick expansion medium. After 12–14 days of culture, plates were screened by ELISA again to isolate virus-specific T cells with high positive signal. The quick expansion protocol was used to expand positive T cells in T25 flasks [32]. After tetramer-guided selection, 1–2 × 10^6^ T cells were used to isolate RNA (RNeasy plus universal mini kit, Qiagen) and were sent to GENEWIZ (Nanjing, China) for ImmunoSEQ.

### Construction of TCR retroviral vector and T cell transduction

TCR nucleotide sequences were synthesized and cloned into the MSGV1-1D3-28Z.1–3 mut (addgene) retroviral vector. The human TCR constant regions were replaced with murine TCR constant regions, and a furin P2A linker was used to connect the TCR chains in the α-β order. Retroviral vectors encoding the target TCRs were first generated by transfecting 293GP packaging cells with pMSGV1-TCR and VSV-G plasmids. Viral vector-containing supernatant were harvested 48 and 72 h post-transfection. PG13-TCR producer cells were established by two consecutive transductions of PG13 cells with harvested viral supernatant. Human PBMC from healthy donors were isolated as mentioned above. CD3^+^ T cells were isolated from PBMCs with Dynabeads™ untouched™ human T cells kit (Life Technologies) and stimulated for 2 days with Dynabeads human T-activator CD3/CD28 (Life Technologies) at a bead to cell ratio of 1:1. Retroviral vectors that produced from PG13-TCR cells were preloaded into RetroNectin-coated 6-well plates (Takara), and activated T cells were transduced with two cycles of spinoculation.

### Flow cytometry

The following fluorescently conjugated antibodies were purchased from BD Biosciences: CD3-BV605/APC/PE (SK7), CD8-APC-H7/FITC (HIT8a), CD4-PE/BV786/APC (SK3), murine TCRβ constant region (mTRBC)-PE-Cy7. Tetramers (HLA-A*02:01-HPV16-E7_11–19_, HLA-A*02:01-EBV-LMP2_356–364,_ HLA-A*02:01-EBV-LMP2_426–434_ and HLA-A*02:01-CMVpp65_495–503_) were purchased from MBL International. Data were acquired with a BD FACSAria™ II flow cytometer (BD Biosciences) and analyzed with FlowJo software (FlowJo).

### T cell in vitro functional assays

For cytokine production assays, 1 × 10^5^ T cells were cocultured with 5 × 10^4^ target cells in 96-well U-bottom plates at 37 °C. For peptide titration experiments, T2 cells were pulsed with different concentrations of peptides overnight. After washing, 5 × 10^4^ peptide-pulsed T2 cells were cocultured with 1 × 10^5^ T cells as mentioned above. Coculture supernatants were harvested after 16–18 h, and IFN-γ concentration was measured by ELISA (Thermo Scientific). Cytotoxicity of T cells was determined by lactate dehydrogenase (LDH) release assays. T cells were cocultured with target cells at the indicated effector-to-target ratios, and LDH in coculture media were quantified according to the manufacturers’ instructions (Pierce LDH cytotoxicity assay kit, Thermo Scientific).

### Treatment of cervical cancer in a xenograft murine model

Animal research protocols were approved by the Animal Experiment Ethics Committee of Shenzhen Second People's Hospital, Shenzhen, Guangdong Province, China. Female (4–6 weeks old) NCG mice (NOD-*Prkdc*^*em26*^*Il2rg*^*em26*^/Nju) were purchased from GemPharmatech (Jiangsu, China). Tumors were initiated by a subcutaneous injection of 2 × 10^6^ CaSki-luc cancer cells on the flank of mice. Twenty-one-days following tumor initiation, T cells (E7 specific TCR-T cells or untransduced T cells were administered by tail vein injection. Mice received adjuvant IL-2 (Jiangsu Kingsley Pharmaceutical Co., Ltd.) 50,000 IU by intraperitoneal injection daily for 3 days beginning immediately after T cell infusion. Tumor load was measured by bioluminescence imaging. Isoflurane-anesthetized animals were imaged using the IVIS system (Xenogen Corp.) 10 min after intraperitoneal injection of 150 mg/kg VivoGlo™ luciferin (Promega). Mice were regularly examined for weight loss, or signs of stress, and euthanized by cervical dislocation according to the preset criteria. At the end of the experiment, Heparinized blood samples were collected by intracardiac puncture under general anesthesia (a single dose of intraperitoneal injection of 250 mg/kg ketamine and 10 mg/kg xylazine). After that, mice were immediately euthanized by cervical dislocation, and tumors and tissues were collected.

### Statistics

Statistical analyses were performed with Prism 5 (GraphPad Software). A two-tailed unpaired *t* test was used to compare the differences between G8 TCR-T cell group and control groups. A *P* value of less than 0.05 was considered significant.

## Conclusions

TCR discovery has played a key role in the development of effective TCR-T cell therapy. The highly diverse TCR repertoire in the human body makes isolation of rare tumor-specific T cells challenging. Incorrect pairing of TCRα and TCRβ chains will lead to the loss of function or specificity; thus, most T cell cloning approaches involve a T cell singularization process. The conventional singularization process involves iterative rounds of stringent limiting dilution and functional tests, whereas most recent methods adopted function-independent singularization, such as multimer-guided single-cell sorting, RT-PCR and subsequent abundant TCR validation. We found that cancer patients are a good source of cloned virus-specific T cells, and stringent singularization was not necessary. T cell clones with strong proliferation potential could outcompete other cells and finally dominate the whole population. Therefore, we combined several nonstringent methods to narrow the repertoire of target T cells in a stepwise manner and finally obtained a single TCR with good properties.

The benefit of isolating TCR from cancer patients has been demonstrated [33]. Patients who had complete responses to immunotherapies, including immune checkpoint blockage [34], vaccines [35], and adoptive T cell transfer [36, 37] would be the best candidates to isolate highly functional TCRs. Although we could not test the expression of viral antigens in these patients and their treatment results are unknown, the PBMCs of these patients showed a higher endogenous T cell response to distinct viruses than normal donors. This is reasonable because HPV and EBV are direct causes of cervical cancer and NPC, respectively, while nearly all people have CMV infection, which may reactivate in an immune-suppressive environment. Due to a relatively high initial frequency of virus-specific T cells in cancer patients, one round of in vitro peptide stimulation is usually sufficient for ELISA, which shortens the timeframe.

Our approach also saves time and has a higher success rate in the cloning process. Considering the statistical nature of the limiting dilution, a stringent dilution rate (0.3–0.5 cell/well) was usually used to guarantee that the final colonies arise from single cells. However, 6–10 times more plates and labor are required to screen the same amount of T cells as using our method. Moreover, because of T-cell exhaustion, some antigen-specific T cells may gradually lose their function or fail to grow in the process of repeated expansion. We found that 3 cells/well consistently gave high positive clones with low background signal. With the increase in cell number per well, the background signal increased as well.

Tetramer-guided cell sorting facilitates the isolation of antigen-specific T cells. However, it requires the frequency of positive cells to be beyond the detection limit of flow cytometry (0.1–1%), and strong tetramer staining is not always correlated with good function. Therefore, we used it in the last polishing step to further purify T cells and retrieve function-exhausted clones.

NGS of bulk T cells has been used to retrieve tumor-specific TCRs based on the abundance of Vα and Vβ [38]. Due to the variation in library construction and PCR with multiple Vα and Vβ primers, the top Vβ may not correctly pair with the top Vα. In addition, it has been reported that up to 30% of T cells express two distinct α chains [39]. To reduce the variation in NGS, we constructed a full-length library with 5’ RACE. In most cases, we obtained only one dominant Vα and Vβ chain (frequency > 90%), and the top Vβ was always correctly paired with the top Vα.

In summary, the modified TCR identification method proposed in this paper not only reduces the time and labor compared with conventional limiting-dilution-based T cell cloning but is also more cost effective than single-cell-based techniques. Our method is readily adoptable to any labs equipped with conventional instruments and can be integrated into an automated process as well. Furthermore, this approach allows T cell clones with potent function and proliferation properties to automatically appear, omitting the tedious validation phase.

## Supplementary Information

**Additional file 1**. **Fig. S1**. The high percentage of virus-specific T cells in the PBMC of cancer patients. (a) PBMCs from a healthy donor and a patient with metastatic melanoma were cocultured with T2 cells pulsed with indicated peptides or DMSO for 16-18 hours, respectively. The release of IFN-γ in the cocultured supernatant was detected by ELISA. (b) PBMC from a healthy donor and a patient with NPC were stained with EBV-LMP2 tetramer, and the cells were gated on tetramer-positive cells (SSC × PE). **Fig. S2**. The CDR3 sequences of isolated virus-specific T cell clones.

## Data Availability

All data generated or analysed during this study are included in this published article.

## Abbreviations

TCR: T cell receptor
EBV: Epstein-Barr virus
HPV: Papillomavirus
KSHV: Kaposi’s sarcoma-associated herpesvirus
MCV: Merkel cell polyomavirus
HBV: Hepatitis B virus
HTLV-1: Human T lymphotropic virus type-1
HCV: Hepatitis C virus
HIV: Human immunodeficiency virus
ACT: Adoptive T-cell therapies
TILs: Tumor-infiltrating lymphocytes
PBMCs: Peripheral blood mononuclear cells
GBM: Glioblastoma multiforme
