# Supplementary material for: An efficient method to identify virus-specific TCRs for TCR-T cell immunotherapy against virus-associated malignancies
Source: BMC Immunol. 2021 Sep 28;22:65. doi: 10.1186/s12865-021-00455-3 (PMC8480097; doi:10.1186/s12865-021-00455-3)
Supplement: Supplementary file 1 — Additional file 1. Fig. S1. The high percentage of virus-specific T cells in the PBMC of cancer patients. (a) PBMCs from a healthy donor and a patient with metastatic melanoma were cocultured with T2 cells pulsed with indicated peptides or DMSO for 16-18 hours, respectively. The release of IFN-γ in the cocultured supernatant was detected by ELISA. (b) PBMC from a healthy donor and a patient with NPC were stained with EBV-LMP2 tetramer, and the cells were gated on tetramer-positive cells (SSC × PE). Fig. S2. The CDR3 sequences of isolated virus-specific T cell clones. [file 12865_2021_455_MOESM1_ESM.pdf]

Supple. Fig. 1

a

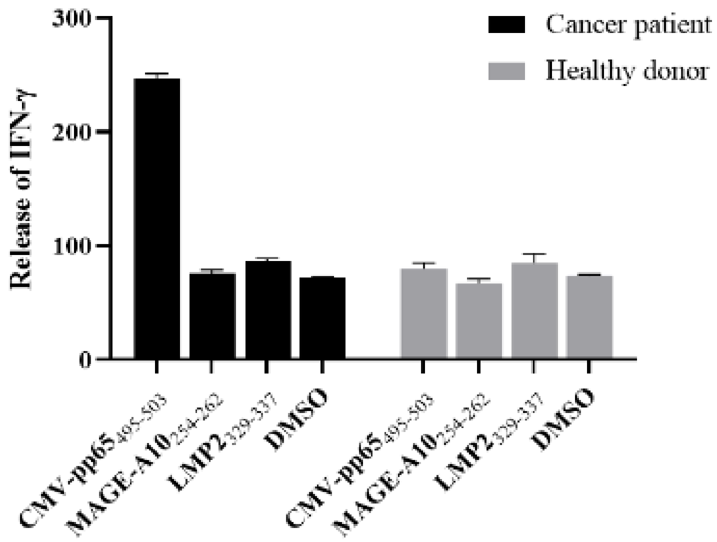

b

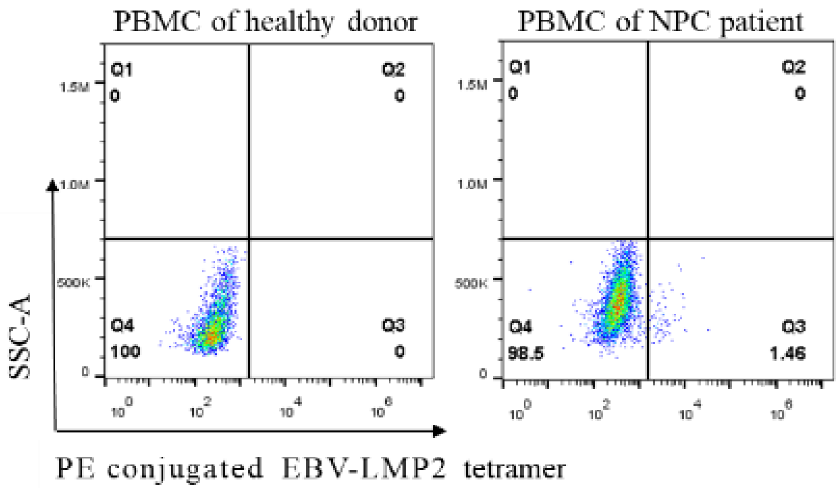

Supple. Fig. 2

TCR clones	CDR3 sequences of beta chains
G8	**C**ASSLAWRGGSYNEQF**F**
A6	**C**ASRDGLAGLSYEQY**F**
A4	**C**ASSSLAGGPNEQF**F**
A12	**C**ASNPQGGNYGYT**F**
C5	**C**ASSYQGGNYGYT**F**
G1	**C**ASSYLGGYYGYT**F**
